# Sciatica

**Published:** 1880-12

**Authors:** 


### SCIATICA.

Three drops of sulphuric ether, mixed with a few drops of water and injected under the skin with a hypodermic syringe, is said to permanently cure this distressing complaint. The remedy should be used every twelve hours till a cure is effected.

